# Assessing Walking Adaptability in Parkinson's Disease: “The Interactive Walkway”

**DOI:** 10.3389/fneur.2018.01096

**Published:** 2018-12-12

**Authors:** Daphne J. Geerse, Melvyn Roerdink, Johan Marinus, Jacobus J. van Hilten

**Affiliations:** ^1^Department of Neurology, Leiden University Medical Center, Leiden, Netherlands; ^2^Department of Human Movement Sciences, Faculty of Behavioural and Movement Sciences, Vrije Universiteit Amsterdam, Amsterdam Movement Sciences, Amsterdam, Netherlands

**Keywords:** Parkinson's disease, walking ability assessment, Interactive Walkway, unconstrained walking, adaptive walking, dual-task walking

## Abstract

**Introduction:** In people with Parkinson's disease (PD) many aspects of walking ability deteriorate with advancing disease. Clinical tests typically evaluate single aspects of walking and to a lesser extent assess more complex walking tasks involving a combination of the three key aspects of walking ability (i.e., generating stepping, maintaining postural equilibrium, adapting walking). The Interactive Walkway allows for assessing more complex walking tasks to address features that are relevant for daily life walking of patients, including adaptive walking and dual-task walking.

**Methods:** To evaluate the expected added value of Interactive Walkway assessments in people with PD, we first evaluated its known-groups validity for outcome measures of unconstrained walking, adaptive walking and dual-task walking. Subsequently, these outcome measures were related to commonly used clinical test scores. Finally, we evaluated the expected added value of these outcomes over clinical tests scores in discriminating people with PD with and without freezing of gait.

**Results:** Interactive Walkway outcome measures showed significant differences between freezers, non-freezers and healthy controls, in expected directions. Most Interactive Walkway outcome measures were not or at best moderately correlated with clinical test scores. Finally, Interactive Walkway outcome measures of adaptive walking slightly better discriminated freezers from non-freezers than clinical tests scores.

**Conclusion:** We confirmed the added value of Interactive Walkway assessments, which provides a comprehensive evaluation of walking ability incorporating features of its three key aspects. Future studies are warranted to examine the potential of the Interactive Walkway for the assessment of fall risk and informing on tailored falls prevention programs in people with PD and in other populations with impaired walking ability.

## Introduction

**Figure 1:**
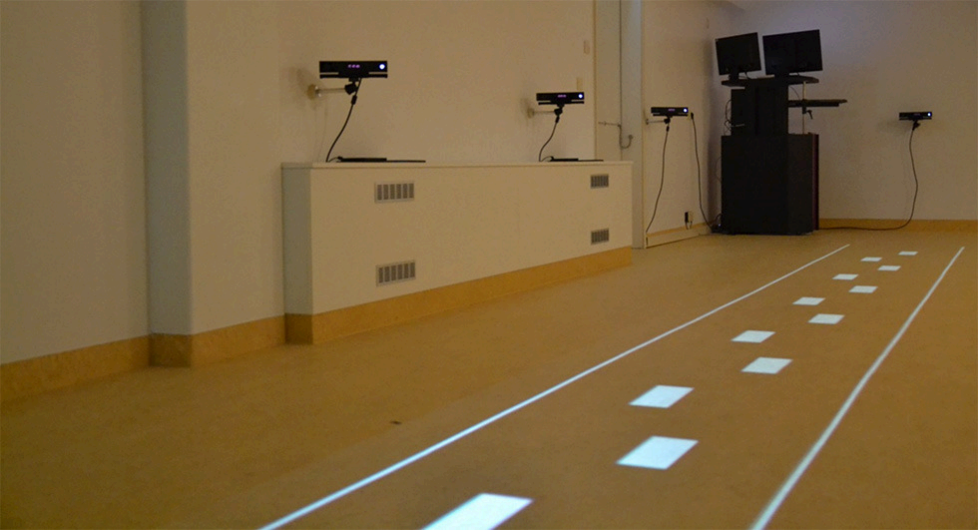
Set-up of the Interactive Walkway with visual context projected on the walkway.

Walking ability is a multifaceted construct which includes the ability to generate stepping, to maintain postural equilibrium, and to adjust walking to meet behavioral goals and environmental demands (1). In Parkinson's disease (PD) these walking ability aspects all deteriorate to some extent with advancing disease. This is evidenced by an inability to generate effective stepping (e.g., freezing of gait [FOG]), a reduced ability to adapt walking to environmental circumstances, and a limited ability to combine walking with secondary tasks (2–5). Such impairments in walking ability may contribute to an increased fall risk. This is clearly demonstrated in PD, where most falls are due to FOG, impaired adaptive walking resulting in trips, and limitations in dual-task walking (6, 7). Clinical tests to evaluate gait and balance disturbances in PD typically evaluate single aspects of walking ability (i.e., the ability to generate stepping or to maintain postural equilibrium) and to a lesser extent assess more complex walking tasks (i.e., adaptive walking and dual-task walking) involving a combination of the three key aspects of walking (stepping, equilibrium and adaptation). The Interactive Walkway (IWW; Figure 1) allows for assessing more complex walking tasks to address features that are relevant for daily life walking of patients, which could guide the management of clinical care.

This study aimed to evaluate the expected added value of IWW assessments in people with PD, which includes an assessment of more complex walking tasks. The IWW utilizes multiple external sensors for a validated quick markerless 3D full-body motion registration of unconstrained walking (8). Moreover, the IWW can be used to assess adaptive walking by augmenting the walkway with visual context, such as suddenly appearing obstacles (9), whose location and timing can be controlled based on real-time processed full-body kinematics. Finally, the IWW may be used to assess the ability to combine walking tasks with a secondary task by quantifying dual-task costs of walking and adaptive walking (10). In this study, we first examined the known-groups validity of IWW outcome measures of unconstrained walking, adaptive walking, and dual-task walking to detect differences between people with PD with FOG, people with PD without FOG and healthy controls. Secondly, we compared IWW outcome measures to commonly used clinical tests of gait and balance impairment to identify redundancy and complementarity among and between tests. Thirdly, we examined the expected added value of the IWW over clinical tests in discriminating people with PD with and without FOG.

## Methods

### Subjects

Walking ability was assessed in 30 people with PD and 30 age- and sex-matched healthy controls (Table 1). People with PD and controls were recruited from the outpatient clinic of the Leiden University Medical Center and via advertisement, respectively. People with PD had to meet the UK Parkinson's Disease Society Brain Bank clinical diagnostic criteria (11) and have a Hoehn and Yahr stage of 1–4 (12). In addition, subjects had to be 18 years or older, have command of the Dutch language, be able to stand unsupported for more than 20 s and walk independently. People with PD were evaluated using the Movement Disorder Society version of the Unified Parkinson's Disease Rating Scale motor score (13). The New Freezing of Gait Questionnaire (14) was used to classify people with PD with and without FOG (i.e., based on a score greater than or equal to zero, respectively), leading to the classification of 14 freezers and 16 non-freezers. The Scales for Outcomes in Parkinson's disease—Cognition (15) was administered to assess cognitive abilities, since this scale is sensitive to PD-specific cognitive deficits. People with PD were measured in the ON state. Controls did not suffer from neurological or orthopedic diseases interfering with gait, had normal cognitive function [Montreal Cognitive Assessment score ≥ 23; (16)] and (corrected to) normal vision. All subjects gave written informed consent, and the study was approved by the local medical ethics committee (P15.232).

**Table 1:** Group characteristics of people with Parkinson's disease (all, freezers and non-freezers) and healthy controls.

		**Parkinson's disease**	**Freezer (*n* = 14)**	**Non-freezer (*n* = 16)**	**Control**
Age (years)	mean ± SD	63.1 ± 10.0	61.8 ± 9.6	64.2 ± 10.5	62.9 ± 10.3
Sex	male/female	18/12	10/4	8/8	18/12
Disease duration (years)	mean ± SD	12.2 ± 6.7	14.3 ± 6.8	10.3 ± 6.3	–
Levodopa equivalent daily dose (mg)^a^	mean ± SD	939 ± 771	1258 ± 947	661 ± 441	–
SCOPA-COG [0-43]^*^	mean ± SD	30.4 ± 7.1	28.9 ± 8.0	31.8 ± 6.3	–
MDS-UPDRS motor score [0-132]^**^	mean ± SD	36.9 ± 18.0	41.4 ± 20.3	32.9 ± 15.3	–
Hoehn and Yahr stage [1–5]^^**^, *a*^	mean ± SD	2.3 ± 0.7	2.6 ± 0.7	2.0 ± 0.5	–
NFOGQ [0-24]^**^	mean ± SD	–	19.9 ± 5.0	0	–
MOCA [0-30]^*^	mean ± SD	–	–	–	27.7 ± 1.4

*SCOPA-COG, Scales for Outcomes in Parkinson's disease – Cognition; MDS-UPDRS, Movement Disorder Society version of the Unified Parkinson's Disease Rating Scale; NFOGQ, New Freezing of Gait Questionnaire; MOCA, Montreal Cognitive Assessment*.

* *Higher scores represent better outcomes*.

** *Higher scores represent worse outcomes*.

a *Significant difference between freezers and non-freezers (p < 0.05)*.

### Experimental Set-Up and Procedure

We used clinical tests of gait and balance impairment that have previously been suggested or recommended for use in people with PD (17). Two tests assessed mobility: the Timed-Up-and-Go test and the 10-m walking test at comfortable and maximum walking speed. Longer completion times indicate poorer mobility. The Tinetti Balance Assessment has two sections that evaluate gait and balance performance of which the combined score was used in this study (higher scores indicate a better performance). Two other balance tests were administered: the 7-item Berg Balance Scale, to measure static and dynamic balance, and the Functional Reach Test, to determine the maximal reaching distance (higher scores indicating a better balance). The order of these clinical tests was randomized.

**Figure 2:**
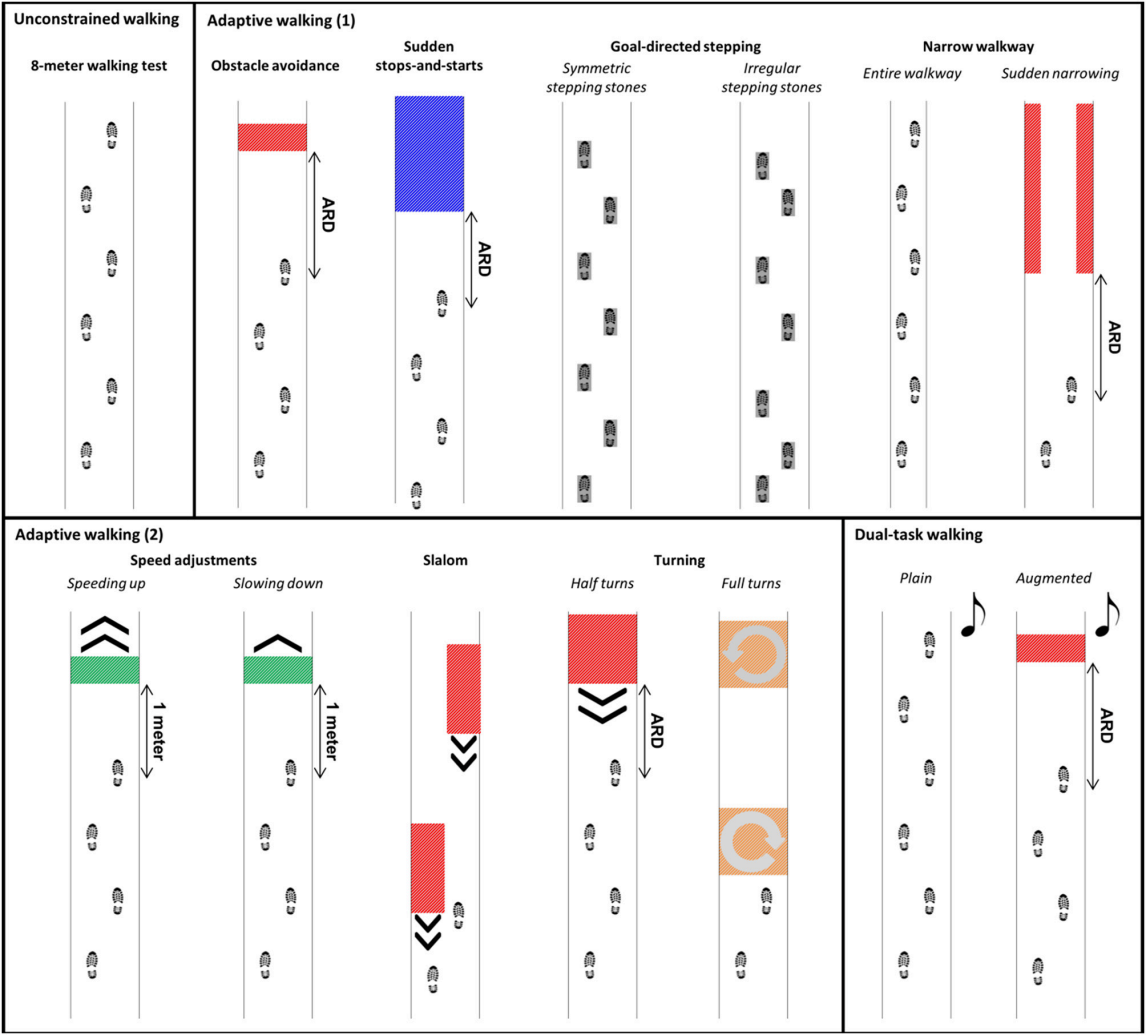
Schematic representation of the Interactive Walkway assessment, including unconstrained walking, adaptive walking, and dual-task walking. The available response distance (ARD) of the suddenly appearing obstacles and cues was patient-tailored to yield a similar response time.

The IWW was used to assess unconstrained walking, adaptive walking and dual-task walking (cf. Figure 2; see Supplementary Video and Table 2 for more details). Full-body kinematics was obtained using four spatially and temporally integrated Kinect v2 sensors, which allows for a quick markerless assessment of walking. The sensor set-up was based on a validated IWW set-up (8, 9), with improved inter-sensor distances following recommendations of Geerse et al. (18) (Figure 1). The sensors were positioned at a height of 0.95 m alongside a walkway of 8 by 0.75 m. The first three sensors were placed frontoparallel (i.e., with an angle of 70° relative to the walkway direction) with a distance of 1.2 m from the left border of the walkway. The last sensor was positioned frontally at the end of the walkway, since this will minimize orientation-based biases. The first sensor was positioned at 3 m from the start and the other sensors were placed at inter-sensor distances of 2.1 m (Figure 1). The IWW was equipped with a projector (EPSON EB-585W, ultra-short-throw 3LCD projector) to augment the entire walkway with visual context. The coordinate systems of the sensors and the projector were spatially aligned using a spatial calibration grid. IWW data were sampled at 30 Hz using custom-written software utilizing the Kinect-for-Windows Software Development Kit (SDK 2.0). Unconstrained walking was assessed with an 8-m walking test. Adaptive walking was assessed with obstacle avoidance, sudden stops-and-starts, goal-directed stepping (symmetric and irregular stepping stones), narrow walkway (entire walkway and sudden narrowing), speed adjustments (speeding up and slowing down), slalom and turning (half and full turns). Dual-task walking was assessed in plain and augmented walking environments by adding an auditory Stroop task in which the words high and low were pronounced at a high or low pitch (i.e., congruent and incongruent stimuli) to the 8-m walking test and obstacle-avoidance task, respectively. Subjects had to respond with the pitch of the spoken word. The IWW assessment contained 36 trials (Table 2). Subjects were instructed to complete each trial at a self-selected walking speed, while also responding to the Stroop stimuli in case of dual-task walking. Figure 2 presents a schematic representation of the IWW assessment.

**Table 2:** Interactive Walkway tasks and outcome measures of unconstrained walking, adaptive walking, and dual-task walking.

		**Trials**	**Level of difficulty**	**Characteristics**	**Outcome measure**	**Unit**	**Calculation**
**UNCONSTRAINED WALKING**							
8-m walking test		2		Walking at self-selected walking speed.	Walking speed	cm/s	The distance traveled between the 0 and 8-m line on the walkway divided by the time, using the data of the spine shoulder.
					Step length	cm	The median of the differences in the anterior-posterior direction of consecutive step locations.
					Stride length	cm	The median of the differences in anterior-posterior direction of consecutive ipsilateral step locations.
					Step width	cm	The median of the absolute mediolateral difference of consecutive step locations.
					Cadence	steps/min	Calculated from the number of steps in the time interval between the first and last estimate of foot contact.
					Step time	s	The median of the time interval between two consecutive instants of foot contact.
					Stride time	s	The median of the time interval between two consecutive ipsilateral instants of foot contact.
**ADAPTIVE WALKING**							
Obstacle avoidance		5	ART = 1 s (three trials) ART = 0.75 s (two trials)	Avoiding suddenly appearing obstacles.	Obstacle-avoidance margins	cm	The distance of the anterior shoe edge (trailing limb) and posterior shoe edge (leading limb) of the step locations to corresponding obstacle borders during obstacle crossing.
					Success rate	%	Number of successfully avoided obstacles divided by the number of obstacles presented times 100%.
Sudden stops-and-starts		5	ART = 1 s (three trials) ART = 0.75 s (two trials)	Stop behind the suddenly appearing stop cues and start walking as soon as the cues disappear.	Sudden-stop margins	cm	The minimum distance of the anterior shoe edge to the corresponding stop cue border during the period in which the cue was visible.
					Success rate	%	Number of successful stops divided by the number of stop cues presented times 100%.
					Initiation time	s	The time between disappearance of the stop cue and the moment of first foot contact.
Goal-directed stepping	SSS ISS	3 2	Average SL 75% average SL 125% average SL 25% variation in SL left and right 50% variation in SL left and right	Stepping as accurately as possible onto the shoe-size-matched stepping stones.	Stepping accuracy	cm	The standard deviation over the signed deviations between the center of the stepping target and the center of the foot at corresponding step locations. The center of the foot was determined using the average distance between the ankle and the middle of the shoe-size-matched targets of the calibration trials (Appendix B).
					Normalized walking speed	%	Walking speed divided by walking speed of the 8MWT times 100%.
Narrow walkway	EW SN	2 1	WW = 1.5*SW+FW WW = SW+FW ART = 1 s, WW = 1.5*SW+FW	Walking between the lines of the walkway or between the blocks of the suddenly narrowing walkway.	Success rate	%	Number of steps inside the walkway or the sudden narrowing walkway divided by the total number of steps taken times 100%.
					Normalized walking speed	%	Walking speed divided by walking speed of the 8MWT times 100%.
					Normalized step width	%	Step width divided by the imposed step width times 100%.
Speed adjustments	SU SD	2 2	120% SSWS 140% SSWS 80% SSWS 60% SSWS	The subject has to follow a speed cue appearing 1 m in front of the subject at the imposed speed.	Success rate	%	The percentage of the time spend walking faster (or slower) than the imposed speed minus (or plus) 20% during the period in which the speed cue was visible.
					Normalized walking speed	%	Walking speed divided by the imposed walking speed times 100%.
Slalom		2	Symmetric distance between obstacles Variable distance between obstacles	Walking around the moving obstacles that approach the subjects with a speed of 50% SSWS.	Success rate	%	Number of successfully avoided obstacles divided by the number of obstacles presented times 100%.
					Normalized walking speed	%	Walking speed divided by walking speed of the 8MWT times 100%.
Turning	HT	2	ART = 3 s ART = 2 s	When a turning cue approaches the subject with a speed of 100% SSWS, the subject has to turn and walk back to the start.	Success rate	%	Number of successful half turns divided by the number of half turns times 100%.
					Turning time	s	Time within the turning square (for full turns) or time from appearance of the turning cue till moment walking direction was reversed (for half turns), using the data of the spine shoulder.
	FT	1		In the two presented squares the subject has to make a full turn as fast and safe as possible in the direction of the arrow.			
**DUAL-TASK WALKING**							
Plain		2		Walking at self-selected walking speed while also performing a dual task. The dual task was an auditory Stroop task.	Normalized walking speed	%	Walking speed divided by walking speed of the 8MWT times 100%.
					Success rate dual task	%	Number of correct responses divided by the number of stimuli given times 100%.
Augmented		5	ART = 1 s (three trials) ART = 0.75 s (two trials)	Avoiding suddenly appearing obstacles and while also performing a dual task. The dual task was an auditory Stroop task.	Normalized success rate	%	Obstacle avoidance success rate divided by success rate of the obstacle avoidance task times 100%, excluding subjects that had an obstacle-avoidance success rate of 0% at baseline.
					Success rate dual task	%	Number of correct responses divided by the number of stimuli given times 100%, excluding subjects that had an obstacle-avoidance success rate of 0% at baseline.
**Total**		**36**					

*SSS, symmetric stepping stones; ISS, irregular stepping stones; EW, entire walkway; SN, sudden narrowing; SU, speeding up; SD, slowing down; HT, half turns; FT, full turns; ART, available response time; SL, step length; WW, walkway width; SW, step width; FW, foot width; SSWS, self-selected walking speed of unconstrained walking, 8MWT, 8-m walking test*.

Half of the subjects started with the block of clinical tests, the other half with the IWW assessment. With regard to the latter, subjects always started with the 8-m walking test, allowing us to adjust the settings of the adaptive walking tasks to one's own gait characteristics in an attempt to obtain a similar level of difficulty for each subject (see Table 2). For example, available response times for suddenly appearing obstacles were controlled by self-selected walking speed during the 8-m walking test and available response distance (ARD in Figure 2). Subsequently, plain dual-task walking was performed, preceded by a familiarization trial in which the dual task was practiced while sitting. The remaining IWW tasks were randomized in blocks (Table 2).

### Data Pre-processing and Analysis

Data pre-processing followed Geerse et al. (8, 9), as detailed in the Supplementary Material. In total, 12 trials (1.1% of all trials) were excluded since subjects were not able to perform the tasks or trials were not recorded properly (i.e., incorrect recording or not all sensors were able to track the subject). These trials only concerned people with PD. The IWW outcome measures of unconstrained walking, adaptive walking and dual-task walking were calculated from specific body points' time series, estimates of foot contact and foot off, and step locations, as detailed in Table 2 and the Supplementary Material. The average over trials per IWW task per subject was calculated for all outcome measures (Table 2).

### Statistical Analysis

IBM SPSS Statistics for Windows, version 24 (IBM Corp., Armonk, N.Y., USA) was used to perform the statistical analyses. With regard to the known-groups validity we examined the effect of group (i.e., freezer, non-freezer or control) on clinical test scores and IWW outcome measures of unconstrained walking, adaptive walking and dual-task walking using one-way ANOVAs or the Kruskal-Wallis test if the assumption of normality was violated (i.e., significant Shapiro-Wilk test). For one-way ANOVAs, the assumption of homogeneity of variance was checked using the Levene's test. If significant, the Welch test was used and main effects were examined using Games-Howell *post-hoc* tests. Otherwise, main effects were examined with Least Significant Difference *post-hoc* tests. For the Kruskal–Wallis test, main effects were examined using multiple Mann–Whitney tests. Effect sizes were quantified with omega squared (ω^2^) for one-way ANOVAs and eta squared (η^2^) for Kurskal–Wallis tests. There was no correction for multiple comparisons due to the explorative character of the study and given the dependency between the outcome measures.

Pearson's correlation coefficients were determined between clinical test scores and IWW outcome measures for people with PD only. Absolute correlations between 0-0.499, 0.500-0.699, 0.700-0.899, and 0.900-1.000 were regarded as low, moderate, high and very high correlations, respectively (19).

Stepwise discriminant analyses were conducted to determine the added value of IWW outcome measures over clinical test scores in discriminating freezers from non-freezers, using Wilks' lambda method (entry = 3.84 and removal = 2.71) in four different models. Predictor variables were clinical test scores (model 1), IWW gait characteristics of unconstrained walking (model 2), IWW outcome measures of adaptive walking (model 3) and IWW outcome measures of dual-task walking (model 4; Table 2). Subjects were only included if they had values for all possible predictor variables. Three not highly correlated predictor variables with the highest effect sizes for the comparison between freezers and non-freezers were selected per model. All models were cross-validated using the leave-one-out method [i.e., each subject is classified by a discriminant function which is based on all subjects except itself; (20)]. The accuracy (i.e., proportion of correctly classified freezers and non-freezers) of discriminant models and cross-validated discriminant models was determined. Furthermore, exact McNemar's tests were performed to establish if one model significantly outperformed the others.

## Results

### Known-Groups Validity

As expected, freezers performed significantly worst, non-freezers performed in-between, and matched controls performed best on almost all assessments (i.e., clinical tests, unconstrained walking and adaptive walking; Table 3). There was one exception; freezers had significantly better stepping accuracies than non-freezers on the goal-directed stepping task with symmetric stepping stones. No significant group differences were found for IWW outcome measures of dual-task walking.

**Table 3:** Means, standard deviations and between-groups statistics of clinical test scores and Interactive Walkway outcome measures of unconstrained walking, adaptive walking, and dual-task walking for freezers, non-freezers and controls.

			**Freezers**	**Non-freezers**	**Control**	**Between-groups**		
			**mean ± SD**	**mean ± SD**	**mean ± SD**	**statistics**	***p*-value**	**Effect size**
**CLINICAL TESTS**								
Timed-Up-and-Go test	Time (s)^b^		12.3 ± 6.9	8.5 ± 3.2	7.4 ± 2.2	*H*_2_ = 6.02	0.049	0.102
10-m walking test	Time (s)^a^^,^^b^	CWS	9.3 ± 2.0	8.0 ± 1.7	7.3 ± 1.0	*H*_2_ = 9.77	0.008	0.166
	Time (s)^b^^,^^c^	MWS	6.9 ± 2.0	6.1 ± 1.2	5.3 ± 0.8	*H*_2_ = 8.66	0.013	0.147
Tinetti Balance Assessment	Score [0–28]^a^^,^^b^^,^^c^		23.8 ± 3.5	26.1 ± 2.3	27.7 ± 0.5	*H*_2_ = 30.69	< 0.001	0.520
7-item Berg Balance Scale	Score [0–14]^b^		10.6 ± 3.1	12.3 ± 2.7	13.3 ± 1.3	*H*_2_ = 10.54	0.005	0.179
Functional Reach Test	Reaching distance (cm)^b^^,^^c^		22.0 ± 9.2	25.7 ± 6.3	29.9 ± 5.6	*F*_(2, 57)_ = 6.98	0.002	0.166
**UNCONSTRAINED WALKING**								
8-m walking test	Walking speed (cm/s)^b^		111.7 ± 26.5	121.4 ± 22.8	134.3 ± 19.0	*F*_(2, 57)_ = 5.46	0.007	0.129
	Step length (cm)^b^		62.8 ± 13.4	70.1 ± 11.1	74.5 ± 9.4	*F*_(2, 57)_ = 5.57	0.006	0.132
	Stride length (cm)^b^		126.1 ± 26.7	140.9 ± 21.9	149.9 ± 18.7	*H*_2_ = 7.90	0.019	0.134
	Step width (cm)		10.7 ± 2.9	9.3 ± 3.1	11.1 ± 2.8	*F*_(2, 57)_ = 2.05	0.138	0.034
	Cadence (steps/min)		112.1 ± 6.9	108.9 ± 13.5	112.3 ± 7.5	*F_(_*_2, 27.8)_ = 0.42	0.659	−0.020
	Step time (s)		0.524 ± 0.036	0.551 ± 0.068	0.526 ± 0.038	*F_(_*_2, 27.4)_ = 0.97	0.391	−0.001
	Stride time (s)		1.052 ± 0.074	1.098 ± 0.140	1.047 ± 0.074	*F_(_*_2, 27.0)_ = 0.91	0.415	−0.003
**ADAPTIVE WALKING**								
Obstacle avoidance	Margins trailing limb (cm)		15.0 ± 8.0	19.1 ± 8.4	19.9 ± 7.3	*F*_(2, 57)_ = 1.95	0.151	0.031
	Margins leading limb (cm)^b^^,^^c^		3.9 ± 9.7	6.3 ± 8.0	12.1 ± 6.1	*F*_(2, 57)_ = 6.70	0.002	0.160
	Success rate (%)^b^^,^^c^		56.4 ± 39.7	67.6 ± 32.0	88.2 ± 11.3	*H*_2_ = 8.59	0.014	0.146
Sudden stops-and-starts	Sudden-stop margins (cm)		−0.9 ± 9.1	4.9 ± 6.2	5.4 ± 9.2	*F*_(2, 57)_ = 2.79	0.070	0.056
	Success rate (%)		62.3 ± 22.2	71.5 ± 13.5	76.8 ± 18.5	*H*_2_ = 4.99	0.083	0.085
	Initiation time (s)		1.522 ± 0.330	1.281 ± 0.108	1.338 ± 0.235	*H*_2_ = 5.17	0.076	0.088
Goal-directed stepping	Stepping accuracy (cm)^a^^,^^c^	SSS	2.5 ± 1.0	3.2 ± 1.0	2.5 ± 0.7	*F*_(2, 57)_ = 4.29	0.018	0.099
	Normalized walking speed (%)^b^	SSS	83.6 ± 17.1	90.6 ± 16.4	96.0 ± 16.5	*H*_2_ = 6.23	0.044	0.106
	Stepping accuracy (cm)	ISS	4.1 ± 1.9	4.8 ± 1.9	3.9 ± 1.0	*H*_2_ = 3.22	0.200	0.055
	Normalized walking speed (%)	ISS	84.0 ± 20.5	88.2 ± 18.8	96.0 ± 15.7	*H*_2_ = 4.77	0.092	0.081
Narrow walkway	Success rate (%)	EW	78.3 ± 25.6	77.2 ± 21.8	84.3 ± 17.4	*H*_2_ = 1.60	0.448	0.028
	Normalized walking speed (%)	EW	86.7 ± 27.8	94.4 ± 11.0	99.0 ± 11.9	*F_(_*_2, 23.1)_ = 1.64	0.216	0.021
	Normalized step width (%)	EW	47.5 ± 22.4	40.4 ± 19.9	37.7 ± 16.1	*F_(_*_2, 55)_ = 0.80	0.455	−0.007
	Success rate (%)	SN	87.1 ± 25.6	83.4 ± 32.0	94.2 ± 13.7	*H*_2_ = 1.21	0.547	0.020
	Normalized walking speed (%)	SN	87.9 ± 21.7	90.5 ± 12.1	92.8 ± 11.8	*H*_2_ = 0.31	0.858	0.005
Speed adjustments	Success rate (%)^b^	SU	61.6 ± 11.5	63.0 ± 15.0	69.7 ± 10.1	*H*_2_ = 6.39	0.041	0.110
	Normalized walking speed (%)	SU	86.8 ± 7.0	87.7 ± 7.8	90.2 ± 6.7	*F_(_*_2, 56)_ = 1.27	0.288	0.009
	Success rate (%)	SD	76.5 ± 4.1	78.7 ± 5.3	79.1 ± 5.2	*F_(_*_2, 56)_ = 1.24	0.297	0.008
	Normalized walking speed (%)	SD	99.3 ± 3.1	97.3 ± 10.2	99.4 ± 2.3	*H*_2_ = 0.54	0.764	0.009
Slalom	Success rate (%)		53.5 ± 16.6	61.7 ± 23.3	55.3 ± 23.0	*F_(_*_2, 56)_ = 0.63	0.539	−0.013
	Normalized walking speed (%)		86.6 ± 24.0	97.1 ± 11.6	94.7 ± 9.6	*F_(_*_2, 23.1)_ = 1.04	0.370	0.001
Turning	Success rate (%)	HT	42.3 ± 40.0	46.9 ± 38.6	65.0 ± 35.1	*H*_2_ = 4.18	0.124	0.072
	Turning time (s)	HT	1.532 ± 0.449	1.453 ± 0.277	1.435 ± 0.251	*H*_2_ = 0.04	0.980	0.001
	Turning time (s)^b^^,^^c^	FT	4.841 ± 2.899	3.322 ± 2.243	2.149 ± 0.961	*H*_2_ = 14.82	0.001	0.256
**DUAL-TASK WALKING**								
Plain	Normalized walking speed (%)		88.5 ± 11.8	79.1 ± 20.0	87.7 ± 9.5	*H*_2_ = 1.93	0.380	0.033
	Success rate dual task (%)		81.6 ± 23.4	94.0 ± 10.1	94.9 ± 12.2	*H*_2_ = 3.92	0.141	0.068
Augmented	Normalized success rate (%)		83.7 ± 50.0	98.0 ± 31.4	97.2 ± 23.9	*H*_2_ = 2.08	0.353	0.038
	Success rate dual task (%)		72.2 ± 26.8	86.1 ± 18.8	91.6 ± 9.2	*H*_2_ = 3.94	0.139	0.072

*CWS, comfortable walking speed; MWS, maximum walking speed; SSS, symmetric stepping stones; ISS, irregular stepping stones; EW, entire walkway; SN, sudden narrowing; SU, speeding up; SD, slowing down; HT, half turns; FT, full turns*.

a *Significant difference between freezers and non-freezers (p < 0.05)*.

b *Significant difference between freezers and controls (p < 0.05)*.

c *Significant difference between non-freezers and controls (p < 0.05)*.

### Correlations Between Outcome Measures

**Figure 3:**
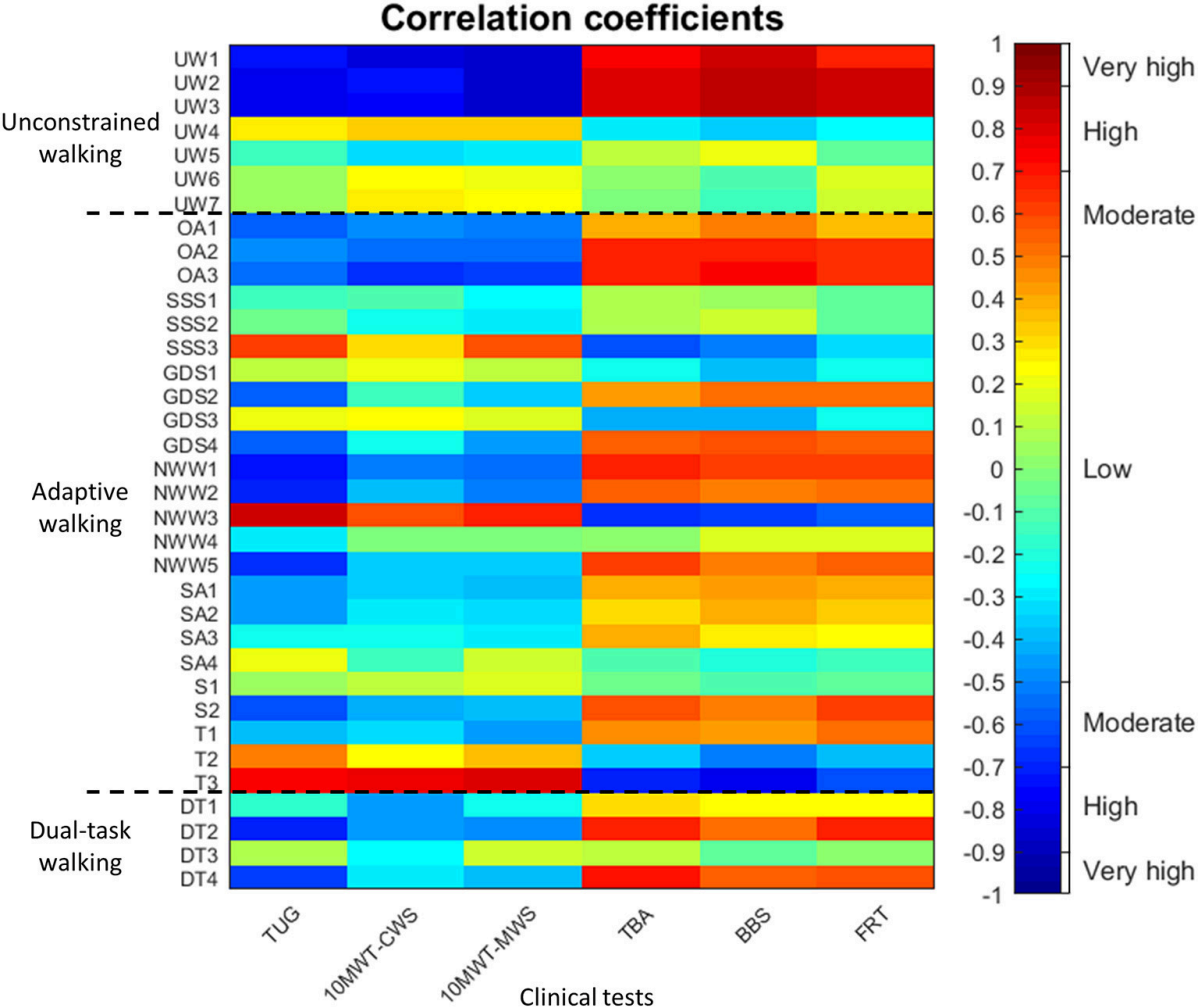
Pearson's correlation coefficients between clinical test scores (x-axis; i.e., Timed Up-and-Go test [TUG], 10-m walking test at comfortable and maximum walking speed [10MWT-CWS, 10MWT-MWS], Tinetti Balance Assessment [TBA], 7-item Berg Balance Scale [BBS] and Functional Reach Test [FRT]) and Interactive Walkway outcome measures (y-axis; i.e., gait characteristics of unconstrained walking [UW1-7], outcome measure of adaptive walking [OA1-3, SSS1-3, GDS1-4, NWW1-3, SA1-4, S1-2, T1-3], and outcome measures of dual-task walking [DT1-4]) in people with Parkinson's disease. The order of the outcome measures on the axes is in agreement with Table 3. The dotted black lines separate the three types of Interactive Walkway tasks (i.e., unconstrained walking, adaptive walking, and dual-task walking). The colorbar provides a visualization of the strength and direction of the correlation.

Of the 42 possible correlations between clinical test scores and IWW gait characteristics, 18 (42.9%) were significant, out of which 17 (40.5%) were high and 1 (2.4%) was moderate (Figure 3). Significant correlations were only found for walking speed, step length and stride length. For IWW outcome measures of adaptive walking, 88 (61.1%) of the possible 144 correlations were significant. Nevertheless, only 9 (6.3%) were high, while 45 (31.3%) were moderate and 34 (23.6%) were low (Figure 3). High correlations were mainly found for turning time of full turns. For IWW outcome measures of dual-task walking, 11 (45.8%) out of the possible 24 correlations were significant, out of which 1 (4.2%) was high, 7 (29.2%) were moderate and 3 (12.5%) were low (Figure 3).

### Discriminant Analyses of Freezers and Non-freezers

For model 1 (clinical tests), group membership (i.e., freezer or non-freezer) was predicted using only the 10-m walking test at comfortable walking speed (*p* = 0.025, Wilks' lambda = 0.791, Canonical correlation = 0.457), the sole predictor variable contributing significantly to the model. Five of ten freezers (50.0%) and 13 of 14 non-freezers (92.9%) were correctly classified. The accuracy of model 1 and its cross validation were both 75.0%. For model 2 (IWW gait characteristics), none of the predictor variables contributed significantly to the model. For model 3 (IWW outcome measures of adaptive walking), group membership was predicted using stepping accuracy on symmetric stepping stones of the goal-directed stepping task and turning time of full turns (*p* = 0.005, Wilks' lambda = 0.598, Canonical correlation = 0.634) such that 7 of 10 freezers (70.0%) and 12 of 14 non-freezers (85.7%) were correctly classified, with an accuracy of 79.2%. The accuracy of the cross-validated model was 70.8%. For model 4 (IWW outcome measures of dual-task walking), none of the predictor variables contributed significantly to the model. The results of an exact McNemar's test demonstrated that there was no statistical significant difference in the proportion of freezers and non-freezers identified with models 1 and 3 (*p* = 0.688).

## Discussion

This study aimed to examine the expected added value of IWW assessments in people with PD, focusing on known-groups validity, relations with clinical test scores and discriminating freezers from non-freezers.

On all clinical tests, freezers scored worst, non-freezers scored in-between and controls scored best (Table 3). These known-groups differences were also found for IWW gait characteristics (Table 3); freezers had significantly lower walking speeds and smaller step and stride lengths than controls, which is in agreement with findings of others using marker-based motion registration systems or the Kinect v2 sensor (21, 22). Significant group differences in expected directions were also observed for IWW outcome measures of adaptive walking (Table 3). As in Caetano et al. (3), both freezers and non-freezers had more difficulty adapting walking to suddenly appearing obstacles than controls as reflected by lower obstacle-avoidance success rates. In line with other studies (23, 24), margins of the leading limb were smaller in freezers and non-freezers, which probably increases their risk of tripping in real life. Furthermore, group differences were found for the goal-directed stepping, speed adjustments and full turns tasks. In general, freezers scored worst, non-freezers in between, and controls best. An interesting exception was stepping accuracy on symmetric stepping stones, where freezers had significantly better stepping accuracies than non-freezers. Irregular stepping stones showed the same trend, although this did not reach significance possibly due to the larger within-groups variations for this task (Table 3). It is well-known that visual cues may lead to considerable improvement in walking of freezers (25). This is likely mediated by a better visual exploration of freezers than non-freezers in terms of gaze fixations to task-relevant information (26), which is known to result in a better stepping performance (27). No significant group differences were found for the sudden stops-and-starts, narrow walkway and slalom tasks. Reasons for the null effect for the narrow walkway tasks could be that step width and tandem gait are typically preserved in people with PD (28), which was corroborated by an absence of between-groups differences in step width in our study. For the other tasks, the cueing effect of the visual context may have confounded potential group differences. Hence, one could consider removing these tasks from adaptive walking assessments in people with PD. For dual-task walking, also no significant group differences were found. An explanation could be that task prioritization varied among subjects, leading to large within-groups variations for the outcome measures of dual-task walking which reduced the likelihood of finding significant between-groups differences. Note that other studies have also demonstrated that there were no differences in dual-task interference for gait characteristics and cognitive tasks between people with PD and controls (29). The added value of dual-task walking in a walking ability assessment in PD is therefore questionable [see also Gaßner et al. (30) and Smulders et al. (10)]. Our study not only confirmed these results, but also showed that quantifiable differences between groups are particularly evident for other aspects of adaptive walking (e.g., obstacle avoidance and goal-directed stepping).

The group differences found for the IWW tasks of unconstrained walking, obstacle avoidance, goal-directed stepping, speed adjustments and full turns imply that these tasks could be used in a comprehensive walking ability assessment with the IWW, incorporating the three key aspects of walking ability. Usually, a combination of the three key walking-ability aspects (i.e., stepping, equilibrium, and adaptation) is needed for a successful task performance. Indeed, for most IWW tasks a combination was required strongly tapping into the aspect of walking adaptability, while adaptation was not or only moderately targeted by commonly-used clinical tests that mainly measure steady-state gait and static balance as evidenced by the low correlations (Figure 3). While high correlations between tests suggest redundancy in information content, low or no correlations suggest that tests contain complementary information. IWW gait characteristics and turning time of full turns correlated highly with clinical tests, addressing mainly aspects of stepping and equilibrium. People with PD seem to experience problems when having to deviate from their normal gait pattern (3), which requires dynamic balance control. Balance problems in people with PD and especially freezers are evident in the current study, demonstrated by large effect sizes for balance tests and full turns. Clinicians mainly focus on gait impairments (31), although dynamic balance control is also of great importance during challenging walking tasks. Therefore, in order to obtain a more comprehensive characterization of a subject's walking ability, both unconstrained and adaptive walking should be assessed, for example with obstacle-avoidance and goal-directed stepping.

This study also aimed to determine the expected added value of the IWW over clinical tests in discriminating freezers from non-freezers. We indeed found that IWW adaptive walking tasks discriminated better than clinical tests, although the added value was somewhat limited and the proportion of freezers and non-freezers identified with model 3 did not differ significantly from model 1. Clinical tests performed slightly worse compared to adaptive walking tasks with regard to the percentage of freezers correctly classified (50.0 vs. 70.0%, respectively). The percentage of non-freezers correctly classified was high for both models (92.9 and 85.7%, respectively). IWW gait characteristics and IWW outcome measures of dual-task walking did not contribute significantly to the discriminant analysis. Although we could discriminate freezers from non-freezers, the freezing phenomenon itself was rarely observed. IWW tasks elicited FOG episodes in only 12 out of 466 (2.6%) trials, concerning five freezers and mostly during tasks that included turning [in agreement with literature; (32)]. Explanations for the limited amount of FOG episodes could be the focused attention due to the specific instructions of the IWW tasks, cueing effects of visual content and the fact that we assessed people with PD during the ON state, while the occurrence of FOG episodes increases during the OFF state.

The latter is also a limitation of this study, since medication may improve gait impairments and could therefore lead to smaller group differences in walking ability. However, we still found significant between-groups differences, which may indicate that the IWW is a sensitive evaluation tool of walking ability. Another limitation is the relatively small sample size of the discriminant analyses (i.e., 10 freezers and 14 non-freezers). We therefore needed to pre-select predictor variables for the models to prevent overfitting, since the smallest group needs to exceed the number of predictor variables. Finally, the significant difference between freezers and non-freezers in disease severity (i.e., Hoehn and Yahr stage; Table 1) might have influenced the results of this study by increasing the group differences of walking ability outcome measures.

In conclusion, the IWW assessment exhibited expected differences between freezers, non-freezers and healthy controls, with most IWW outcome measures reflecting combinations of stepping, equilibrium, and adaptation; key aspects of walking that are addressed separately in most clinical tests. IWW adaptive walking tasks also contributed to a slightly better discrimination of freezers from non-freezers. Hence, it seems fair to conclude that the IWW is of added value in people with PD when assessing walking ability. The IWW tasks of adaptive walking evaluate more complex gait in comparison with clinical tests, which fits an assessment of walking ability in the early stages of PD where ceiling effects can occur. Future studies should examine the responsiveness of the IWW outcome measures on an individual level and in response to levodopa treatment (i.e., by examining differences in walking ability between the ON and OFF state). In addition, since the impairments in walking ability evaluated with the IWW are linked to walking-related falls, future studies are warranted to examine the clinical potential of the IWW for assessing fall risk and informing on tailored falls prevention programs in people with PD or other populations prone to declines in walking ability (e.g., elderly, stroke). Note that the current study is helpful in that regard, by informing on the subtasks and associated outcome measures providing complementary information with a decent between-groups contrast.

## Data Availability Statement

The raw data supporting the conclusions of this manuscript will be made available by the authors, without undue reservation, to any qualified researcher.

## Ethics Statement

All subjects gave written informed consent, and the study was approved by the local medical ethics committee (P15.232).

## Author Contributions

DG conceived and designed the study, acquired the data, analyzed, and interpreted the data and wrote the paper. MR, JM, and JvH conceived and designed the study and wrote the paper. Each of the authors has read and concurs with the content in the final manuscript.

### Conflict of Interest Statement

The authors declare that the research was conducted in the absence of any commercial or financial relationships that could be construed as a potential conflict of interest.

## Supplementary Material

Supplementary Video Video of Interactive Walkway tasks of unconstrained walking, adaptive walking and dual-task walking in a person with Parkinson's disease with dyskinesia. The subject had consented to the making of the video for publication purposes.

Supplementary Material Data pre-processing.
