# Supplementary material for: Assessing Walking Adaptability in Parkinson's Disease: “The Interactive Walkway”
Source: Front Neurol. 2018 Dec 12;9:1096. doi: 10.3389/fneur.2018.01096 (PMC6315126; doi:10.3389/fneur.2018.01096)
Supplement: Supplementary Material — Data pre-processing. [file Data_Sheet_1.pdf]

# *Supplementary Material*

# Assessing walking adaptability in Parkinson's disease: "The Interactive Walkway"

**Daphne J. Geerse**[*]**, Melvyn Roerdink, Johan Marinus and Jacobus J. van Hilten**

*** Correspondence:** Daphne J. Geerse (d.j.geerse@vu.nl)

## 1 Data pre-processing

The Kinect for Windows Software Development Kit (SDK 2.0, www.microsoft.com) provides 3D time series of 25 body points using inbuilt and externally validated human-pose estimation algorithms [1-5]. These body points are: head, neck, spine shoulder, spine mid, spine base and left and right shoulder, elbow, wrist, hand, thumb, hand tip, hip, knee, ankle and foot. For offline data analysis, the 3D positional data for these body points were first pre-processed per Kinect sensor separately. Body points labelled as inferred (i.e., Kinect's human-pose estimation software infers positions when segments are partially occluded for example) were treated as missing values. The body point's time series were linearly interpolated using Kinect's time stamps to ensure a constant sampling frequency of 30 Hz, without filling in the parts with missing values. We removed data points from the time series when they did not meet our stringent requirements for valid human-pose estimation (e.g., a minimum of 15 out of the 25 possible body points should be labeled as tracked, including the head and at least one foot and ankle, without outliers in segment lengths). In addition, a manual check of the data was added to remove errors of the algorithm due to occlusion of the right leg by the left leg. Subsequently, data of the four Kinect sensors were combined by taking for each sample the 3D positions of the body points of a validly estimated human pose. If, for a given sample, more than one sensor contained valid human pose data, the associated body point's 3D positions were averaged for that specific sample.

Body point's time series with more than 50% of missing values were excluded from further analyses. However, percentages of missing data for both groups did not exceed 27.2% with an average of 5.3±1.6% for the body points' time series of interest (i.e., ankles, spine base and spine shoulder). The missing values were interpolated with a spline algorithm. The so-obtained time series were used for the calculation of the Interactive Walkway outcome measures of unconstrained walking, adaptive walking and dual-task walking.

The outcome measures of the Interactive Walkway assessment were calculated from specific body points' time series, estimates of foot contact and foot off and step locations, as detailed in Table 2. Estimates of foot contact and foot off were defined as the maxima and minima of the anterior–posterior time series of the ankles relative to that of the spine base [3,6,7]. Step locations were determined as the median anterior–posterior and mediolateral position of the ankle joint during the single-support phase (i.e., between foot off and foot contact of the contralateral foot; [3,6]). Shoe edges and center of the foot were also needed to calculate several outcome measures. Ankle-to-shoe calibration trials, in which the subject was standing in two shoe-size-matched targets at a position on the walkway in front of the last Kinect, were included to determine the average distance between shoe edges and the ankle.

## 2 References

[1] Clark RA, Pua YH, Oliveira CC, Bower KJ, Thilarajah S, McGaw R, et al. Reliability and concurrent validity of the Microsoft Xbox One Kinect for assessment of standing balance and postural control. Gait Posture (2015) 42(2):210-213.

[2] Dolatabadi E, Taati B, Mihailidis A. Concurrent validity of the Microsoft Kinect for Windows v2 for measuring spatiotemporal gait parameters. Med Eng Phys (2016) 38(9):952-958.

[3] Geerse DJ, Coolen BH, Roerdink M. Kinematic Validation of a Multi-Kinect v2 Instrumented 10-Meter Walkway for Quantitative Gait Assessments. PLoS One (2015) 10(10):e0139913.

[4] Mentiplay BF, Perraton LG, Bower KJ, Pua YH, McGaw R, Heywood S, et al. Gait assessment using the Microsoft Xbox One Kinect: Concurrent validity and inter-day reliability of spatiotemporal and kinematic variables. J Biomech (2015) 48(10):2166-2170.

[5] Xu X, McGorry RW. The validity of the first and second generation Microsoft Kinect™ for identifying joint center locations during static postures. Appl Ergon (2015) 49:47-54.

[6] Geerse D.J., Coolen B.H., Roerdink M. Walking-adaptability assessments with the Interactive Walkway: between-systems agreement and sensitivity to task and subject variations. Gait Posture (2017) 54:194–201.

[7] Zeni J.A., Richards J.G., Higginson J.S. Two simple methods for determining gait events during treadmill and overground walking using kinematic data. Gait Posture (2008) 27(4):710–714.
